# Atypical gaze patterns in autistic adults are heterogeneous across but reliable within individuals

**DOI:** 10.1186/s13229-022-00517-2

**Published:** 2022-09-24

**Authors:** Umit Keles, Dorit Kliemann, Lisa Byrge, Heini Saarimäki, Lynn K. Paul, Daniel P. Kennedy, Ralph Adolphs

**Affiliations:** 1 Division of the Humanities and Social Sciences, California Institute of Technology, Pasadena, USA; 2 Division of Biology and Biological Engineering, California Institute of Technology, Pasadena, USA; 3 Chen Neuroscience Institute, California Institute of Technology, Pasadena, USA; 4 Department of Psychological and Brain Sciences, The University of Iowa, Iowa City, USA; 5 Department of Psychology, University of North Florida, Jacksonville, USA; 6 Faculty of Social Sciences, Tampere University, Tampere, Finland; 7 Department of Psychological and Brain Sciences, Indiana University, Bloomington, USA

**Keywords:** Eye tracking, Autism, Heterogeneity, Individual differences, Videos

## Abstract

**Background:**

Across behavioral studies, autistic individuals show greater variability than typically developing individuals. However, it remains unknown to what extent this variability arises from heterogeneity across individuals, or from unreliability within individuals. Here, we focus on eye tracking, which provides rich dependent measures that have been used extensively in studies of autism. Autistic individuals have an atypical gaze onto both static visual images and dynamic videos that could be leveraged for diagnostic purposes if the above open question could be addressed.

**Methods:**

We tested three competing hypotheses: (1) that gaze patterns of autistic individuals are less reliable or noisier than those of controls, (2) that atypical gaze patterns are individually reliable but heterogeneous across autistic individuals, or (3) that atypical gaze patterns are individually reliable and also homogeneous among autistic individuals. We collected desktop-based eye tracking data from two different full-length television sitcom episodes, at two independent sites (Caltech and Indiana University), in a total of over 150 adult participants (*N* = 48 autistic individuals with IQ in the normal range, 105 controls) and quantified gaze onto features of the videos using automated computer vision-based feature extraction.

**Results:**

We found support for the second of these hypotheses. Autistic people and controls showed equivalently reliable gaze onto specific features of videos, such as faces, so much so that individuals could be identified significantly above chance using a fingerprinting approach from video epochs as short as 2 min. However, classification of participants into diagnostic groups based on their eye tracking data failed to produce clear group classifications, due to heterogeneity in the autistic group.

**Limitations:**

Three limitations are the relatively small sample size, assessment across only two videos (from the same television series), and the absence of other dependent measures (e.g., neuroimaging or genetics) that might have revealed individual-level variability that was not evident with eye tracking. Future studies should expand to larger samples across longer longitudinal epochs, an aim that is now becoming feasible with Internet- and phone-based eye tracking.

**Conclusions:**

These findings pave the way for the investigation of autism subtypes, and for elucidating the specific visual features that best discriminate gaze patterns—directions that will also combine with and inform neuroimaging and genetic studies of this complex disorder.

**Supplementary Information:**

The online version contains supplementary material available at 10.1186/s13229-022-00517-2.

## Background

Autism spectrum disorder (ASD) is widely recognized to be a complex and heterogeneous disorder that has so far defied any simple diagnostic markers or cognitive mechanisms [1]. Yet there is a universal agreement that processing of social stimuli is prominently atypical [2, 3], with reported difficulties encompassing orienting and attention to biological stimuli [4, 5], to processing the features of faces [6–10], to making inferences about the intentions [11], emotions [12–14], and beliefs [15, 16] of other people, and finding social stimuli rewarding [17–21]. Many of these processes can be measured, at least in part, through behavioral choices, verbal ratings, neuroimaging, or eye tracking. Of particular value has been eye tracking, which has the benefit of being relatively unobtrusive and relatively immune to deliberate control, generating a large literature study in ASD [22]. Moreover, eye tracking measures can be obtained in preverbal infants and are thought to reflect early attentional biases that may contribute to the subsequent emergence of cognitive and behavioral components of the autism phenotype [23].

Eye gaze and cognition are intimately related. Where we look is determined by a host of cognitive processes (all of those that determine visual saliency, a broad construct), but eye gaze itself also has a causal effect on cognition [24]. Our gaze influences what we consciously perceive, what we subsequently remember, and what we expect. The detailed fixations spontaneously made onto a face by a given participant directly shape the information that is represented in the brain [25]. Indeed, from the pattern of neural responses to visual scenes it is possible to reconstruct the fixations that people made while they viewed those scenes [26]. Differential eye movement tendencies across participants can be thought of as an endophenotype reflecting substantial genetic effects [27, 28]. Quantifying the gaze patterns in autistic people thus provides an exceptionally rich inventory of how they process the world, and a sensitive window both into genetic predispositions and into phenotypic expression—especially in regard to social behavior.

Multiple factors likely contribute to atypical gaze in ASD. Perhaps, the most robust general finding is that autistic people do not fixate faces typically, including expressive features (eyes, mouth) within faces, although this depends on context [7, 27, 29, 30]. These atypical fixations are thought to reflect atypical social attention, a leading hypothesis in autism research [31, 32]. Another robust finding is that autistic infants have a reduced preference for biological motion stimuli [4, 33–35]. Other theories propose atypical attention to visual stimuli that involve other people’s direction of eye gaze [36], prediction [37], geometric patterns [38], local versus global processing [39], or imitation engagement [40].

Our own work has similarly argued that atypical gaze in ASD is distributed across a broad range of visual features [41]. Specifically, that study quantified gaze onto features of visual stimuli, encompassing low-level features (e.g., contrast), object-level features (e.g., faces), and semantic-level features (e.g., emotions) in a series of images used in computer vision studies [42]. We found that autistic people gaze atypically at images as a function of both low-level and higher-level features. Those differences magnify with time through the trial (across the serial order of fixations onto the image) and include prominent global biases (for instance, autistic people look more at the center of the screen, on average). While we did find a greater effect of semantic-level features than pixel-level features, our prior study [41] suffered a major limitation that is ubiquitous in autism studies: we do not know whether the atypical gaze patterns reflect a temporally stable group difference, or whether they reflect variability within individuals.

Across ASD eye tracking studies, this is a major unanswered question: what portion of the variance in findings can be assigned to between-individual variability, and what portion can be assigned to within-individual variability? In general, there is substantial variability across autistic participants [43–45]. However, there is also variability among participants [46]. Furthermore, these sources of variability may not be static over development or consistent across the wide range of cognitive functioning seen in ASD. In part for this reason, and to ensure our ability to produce valid data on our tasks, we restricted ourselves to adult participants with IQ in the normal range in this study.

Because individual-level reliability is a prerequisite for successful classification and prediction—and important for personalized intervention, we sought to address this question: are gaze patterns from autistic individuals simply noisier and less reliable, or are there stable phenotypic patterns that vary across individuals? The first would suggest specific shared mechanisms that might contribute to ASD (e.g., noise in neural processing), while the second points to heterogeneity, individual differences, and the possibility of subtypes [47]. To optimize generalizability, we collected data across two separate sessions for each participant and tested participants at two different sites (Caltech and Indiana University) using otherwise identical eye trackers and video stimuli.

We tested three competing hypotheses: (1) that gaze patterns of autistic individuals are less reliable or noisier than those of controls, (2) that atypical gaze patterns are individually reliable but heterogeneous across autistic individuals, or (3) that atypical gaze patterns are individually reliable and also homogeneous among autistic individuals.

## Methods

The overall aim of this study was to quantify variability in eye gaze to videos in autistic adults. We aimed to quantify the within-individual variability both across two different sessions of eye tracking across two different video stimuli, as well as the between-individual variability across autistic participants, across control participants, and between the two groups constituted by these individuals. We then interpreted our results considering the three hypotheses stated above.

### Participants

Data used in this study were collected as part of a larger study that included extensive behavioral assessment, neuroimaging, and eye tracking. Here we present only the eye tracking data (collected outside the scanner), together with a subset of the behavioral assessment data. Participants were recruited through flyers distributed at outpatient treatment programs, community support groups, and other community locations (various college campuses, YMCA, etc.). We initially recruited a total of 183 individuals, 67 of whom were adults with a DSM diagnosis of ASD (20 female; 22 tested at the Caltech site) and 116 of whom were typically developing (TD) controls (33 females; 34 tested at the Caltech site). One hundred seventy-six participants watched Episode A (see details of stimuli below; 62 ASD), and 167 participants watched Episode B (59 ASD). We limited our analyses to those 166 participants who watched both Episodes A and B (58 ASD). We subsequently excluded 10 autistic and 3 control participants due to eye tracking data quality (see Eye tracking and Gaze heatmaps sections for details). The final sample consisted of 48 autistic individuals (age 18–41 years, mean = 27.69 years; 17 tested at the Caltech site) and 105 TD controls (age 19–55 years, mean = 27.09 years; 33 tested at the Caltech site) of similar age, sex ratio, and full-scale IQ (Table 1).

**Table 1:** Participants’ demographic information, IQ, and ADOS scores (for ASD group)

	TD mean (SD)	ASD mean (SD)	TD/ASD min–max	Test	*p*
Sample	105	48			
Fraction male	70.5%	79.2%		χ^2^ = 0.864	0.353
Age at experiment (years)	27.09 (7.29)	27.69 (5.37)	19–55/18–41	t(151) = − 0.501	0.617
Full-scale IQ**	110.01(10.20)	111.38 (14.98)	85–136/84–150	t(140) = − 0.637	0.525
ADOS CSS-Overall	–	7.04 (1.98)	–/3–10	–	–
AQ	15.70 (6.41)	28.06 (7.67)	4–40/10–48	t(151) = − 10.314	< 0.001

*TD* typically developing control group, *ASD* autism spectrum disorder group. ADOS CSS-Overall: calibrated severity scores, which were generated from the Hus and Lord [48] algorithm (ASD group only). AQ: the Autism Spectrum Quotient [49]. T tests were two-tailed and unpaired, assuming equal variance. χ^2^ was used to test for independence of the fraction of males between two groups

**10 TD and 1 autistic individuals with missing IQ scores were excluded from this comparison and *t* test

ASD group enrolled at Caltech met the following criteria: (a) had a prior diagnosis of ASD made by a qualified clinician (M.D. or Ph.D.), (b) ASD diagnosis was confirmed through a clinical interview with a participant (and parent, if available) conducted by L.K.P. (who is a licensed clinical psychologist), and (c) scores from ADOS-2 Module 4 administered at Caltech were either at or above the ASD-diagnostic cutoff on at least two components of the original algorithm and within 1 point of the cutoff for the third component or within 1 point of the total score diagnostic cutoff using the Hus and Lord [48] algorithm (i.e., Social Affect + Restricted Repetitive Behaviors >  = 7). Autistic individuals were not excluded due to comorbid diagnoses of depression, anxiety, or ADHD, but were excluded if the total score on Beck Depression Inventory II fell in the moderate–severe range (25 +).

ASD group enrolled at Indiana University had a prior community diagnosis of ASD or Autism, Asperger’s Syndrome, or Pervasive Developmental Disorder-Not Otherwise Specified (PDD-NOS). The ADOS-2 Module 4 was administered at Indiana University by research-reliable administrators; scores were within 1 point of the total score diagnostic cutoff using the revised scoring algorithm (Hus and Lord algorithm; SA + RRB >  = 7, as indicated above for Caltech). We excluded participants taking antipsychotic medication.

The frequency of ASD symptoms was measured in both groups using the Autism Quotient (AQ) [49]. In the ASD group, autism symptom severity was indexed with Overall Calibrated Symptom Severity (CSS-Overall) derived from the ADOS-2 using the Hus and Lord algorithm [48].

### Stimuli

Participants freely watched two episodes of the television sitcom “The Office” (NBC Universal, originally aired in 2005). Episode 1 of Season 1 (22 min., called Episode A here) was viewed in three separate parts, one shortly after the next one (part 1, 6 min. 58 s.; part 2, 8 min. 30 s.; part 3, 6 min. 28 s.), with no task between parts. Episode 4 (21 min., called Episode B here) was shown in a different testing session paused at times and participants were briefly asked to verbally respond to social comprehension questions, as described in [43], but gaze data during these pauses were excluded from the analyses performed in this study. Both episodes were viewed on the same day separated by a break.

To ensure that familiarity with the stimuli did not influence our results, all participants also completed a brief 9-point questionnaire asking about prior familiarity with each of these two episodes (with 1 meaning “not at all” and 9 meaning “very much”). These data were used to split participants into those who were familiar (rating > 5) or unfamiliar (rating < 5) with each episode. Questionnaire data were missing for one autistic and two control participants in Episode A and for two autistic and six control participants in Episode B. These participants were excluded from this familiarity analysis. For Episode A, 17 (24) autistic individuals and 50 (47) TD controls were familiar (unfamiliar) with the episode. For Episode B, 13 (32) autistic individuals and 39 (52) TD controls were familiar (unfamiliar) with the episode. This familiarity information was then used to verify that there were no significant differences in any of our reported results between familiar and unfamiliar groups, either when confined to the ASD group or to the TD group.

### Eye tracking

Participants were comfortably seated in front of a Tobii TX300 eye tracker at approximately 65 cm from the screen (a movable 23″, 1920 × 1080 widescreen monitor). The eye tracker provided calibrated gaze data at 300 Hz (0.4° spatial resolution). Gaze data were collected at 120 Hz at the Caltech site for 12 participants (2 ASD) because of a user error. As gaze data were down-sampled to the video frame rate (24 Hz) before our analyses (see the next section for details), this difference in sampling frequency was compensated across participants. We focused our analyses on the raw gaze data; analyses using derived fixations yield similar results. Prior to starting the videos, participants carried out a 9-point calibration on the screen, followed by a 9-point validation in which the gaze error to the 9 calibrated locations was quantified; the 9 target dots spanned the full extent of the screen. For Episode A, the calibration–validation procedure was repeated prior to viewing each of the three parts. For Episode B, the procedure was completed once at the very beginning of the episode. Quantitative accuracy results were immediately displayed to the experimenter, who would adjust the screen and/or participant and redo the calibration–validation procedure if any of the points had > 1.5° of error. A computer error resulted in the loss of the validation data, so further quantitative assessment of validation accuracy is not possible. We excluded nine autistic and two TD control individuals from our analyses as their gaze data were missing (either missing data points from the eye tracker or gaze out of the stimuli presentation monitor) at more than half of the video watching duration in one or both episodes.

**Fig. 1:**
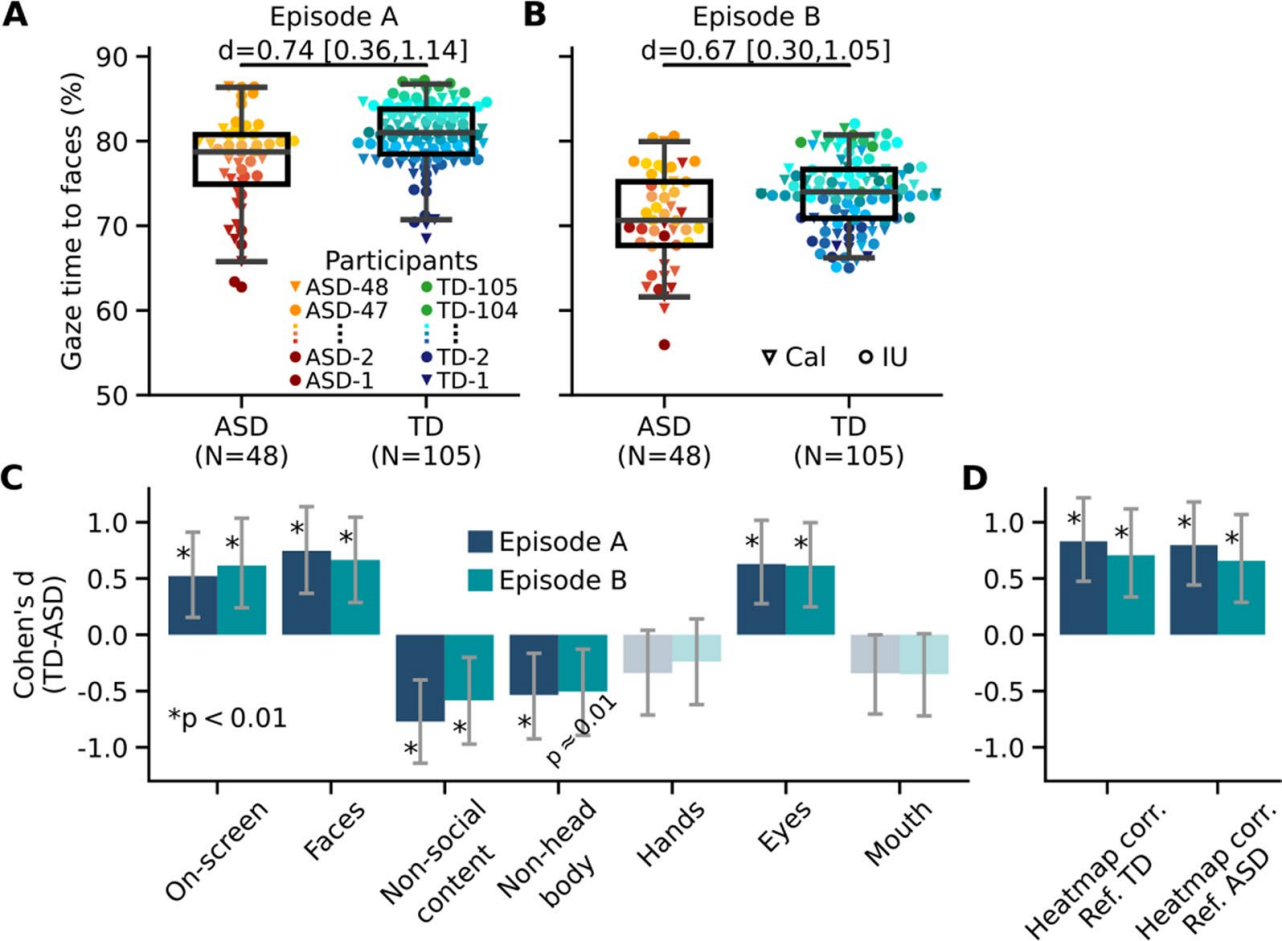
Eye tracking demonstrates reliable gaze differences to features of videos. **A** ASD versus TD comparison in their percentage of total gaze time to faces in the stimulus video Episode A. Error bars span the 2.5–97.5th percentiles, boxes span the 25th to 75th percentiles, and horizontal black lines indicate medians. Effect size of the difference between groups (Cohen’s *d*) is shown on top of the plot. Individuals are denoted by distinct red-yellow spectrum colors based on their percentage of gaze time to faces in Episode A and the same participant-wise colors were used for Episode B (also for Fig. 2). Inverted triangles: participants tested at Caltech; circles: participants tested at Indiana University. **B** Same plot as in **A**, but for data from video Episode B. **C** Effect size of the differences between groups in their percentage of total gaze time to several areas of interest in two separate videos. Bar heights show Cohen’s *d* and error bars show their bootstrap confidence intervals. Saturated colors, asterisks, and *p* values show the statistical significance of Cohen’s *d* (*p* < 0.05, assessed with bootstrap tests, and corrected for multiple comparisons via false discovery rate); desaturated colors show nonsignificant differences. **D** Effect size of the differences between groups in their average correlation with reference gaze heatmaps created by either combining all TD controls (Ref. TD) or all autistic individuals (Ref. ASD). Same format as **C**

**Fig. 2:**
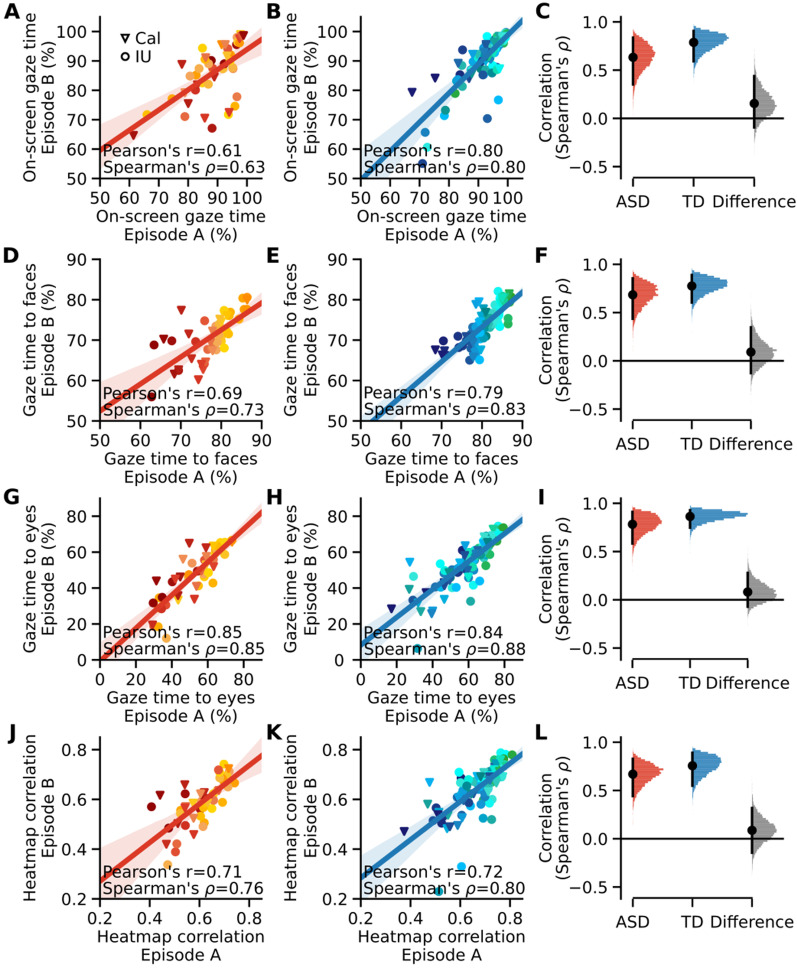
High within-individual reliability in ASD. **A**, **B** Individual participants’ percentage of on-screen gaze time is plotted using data from two separate videos. Individual participants are denoted by red-yellow (ASD) or dark–light blue (TD) spectrum colors that encode the percentage of gaze time to faces in Episode A (panels **D**, **E**; as in Fig. 1A, B) and the same participant-wise color codes were used for other panels (panels **A**, **B**, **G**, **H**, **J**, and **K**). Triangular (circular) markers indicate participants from Caltech (IU) site. Line: Pearson’s correlation and bootstrapped CI are depicted for visualization purposes, but Spearman’s correlation was used to assess reliability in gaze patterns. **D**, **E** Individual participants’ percentage of gaze time to faces is plotted from two separate videos. **G**, **H** Individual participants’ percentage of gaze time to eyes is plotted from two separate videos. **J**, **K** Individual participants’ average gaze heatmap correlation with TD reference gaze heatmaps. **C**, **F**, **I, L** Sampling analysis based on 10-min epoch from the videos and bootstrap resampling of individual participants

**Fig. 3:**
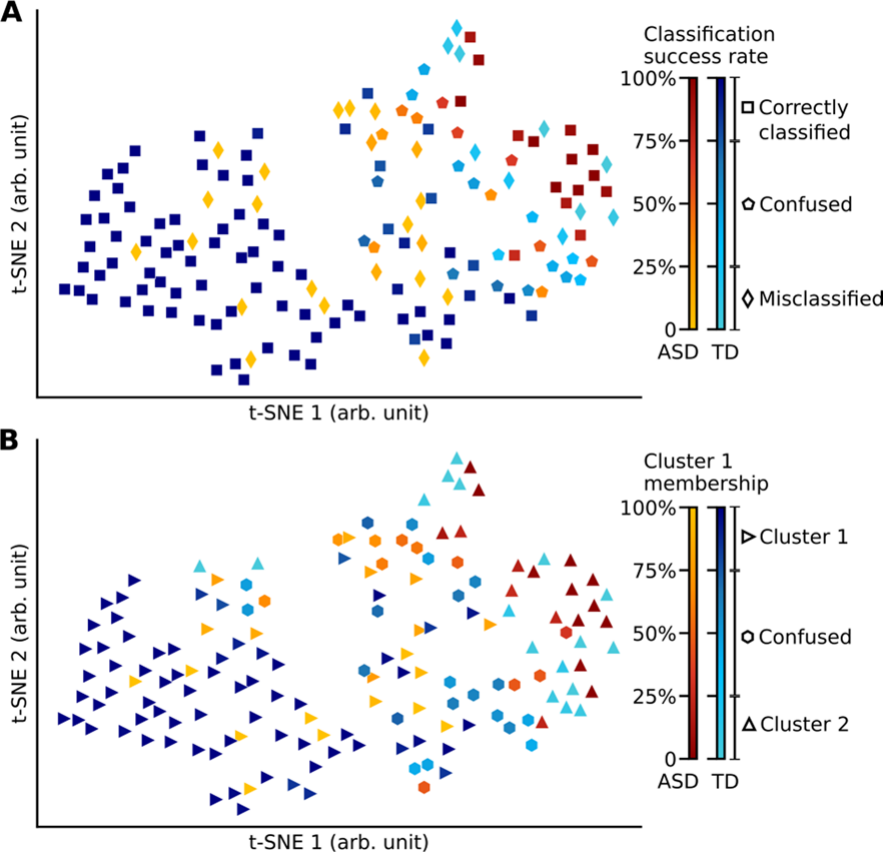
Classification and clustering of participants based on the similarity of gaze patterns. **A** Correctly classified and misclassified participants across cross-validation iterations of a Gaussian Naive Bayes classifier. Color bar encodes the frequency at which an individual participant was classified correctly. Participants denoted by square (diamond) markers were correctly classified (misclassified) more than 75% of the time across iterations. Participants shown with pentagon markers were classified with a frequency lower than 75% (i.e., confused as autistic or TD control in different iterations). Red-yellow (dark-light blue) spectrum colors depict autistic (TD control) participants. The four-dimensional gaze features space used to build the classifier was projected onto a two-dimensional t-SNE space here for visualization purposes only. **B** Unsupervised clustering of participants into subgroups using a Gaussian mixture model procedure. The clustering procedure automatically identified two groups of participants (indicated as Cluster 1 and 2). Color bar encodes the frequency at which an individual participant was assigned to Cluster 1. Participants denoted by right- (up-) pointing triangle markers were assigned to Cluster 1 (Cluster 2) more than 75% of the time across iterations. Participants shown with hexagon markers were assigned with a frequency lower than 75% (i.e., confused as Cluster 1 or 2 in different iterations)

In analyses described below, eye tracking data were used in two main ways: (i) data from two episodes were analyzed separately to compare gaze patterns across the two episodes (e.g., Figs. 1 and 2—all panels other than panels C, F, I, L), (ii) data from two episodes were combined into a single 43-min dataset and analysis procedures were performed on the combined dataset together with data sampling procedures to investigate how identified gaze patterns change with changing video epoch duration and to estimate confidence interval and statistical significance of computed gaze metrics (e.g., Figs. 2C, F, I, L and 3).

### Automatic segmentation of frames to areas of interest (AOIs)

Human body parts in each frame of videos were detected by using a pre-trained neural network model provided in the DensePose module in the Detectron2 deep learning software [50, 51]. Twenty-four different body parts provided by the DensePose model were merged to obtain three main parts of the body for our interest: the head area, hands area, and other body parts (see Additional file 1: Fig. S1). The regions within a frame where no body parts were detected were taken as non-social content. Within each frame, face areas and five facial keypoints (two eyes, nose, and two sides of the mouth) were detected by using the RetinaFace face detector model provided in the InsightFace deep face analysis toolbox [52, 53]. The five keypoints were used to automatically define eyes and mouth areas by following these steps: (i) outline the smallest bounding box that encloses the five keypoints of a face, (ii) outline a medium bounding box that has corners at midpoints between the corners of the small bounding box defined in (i) and the corners of a larger bounding box provided by the face detector method that encloses the total face area, (iii) the total area in the medium bounding box defined in (ii) was divided into two parts based on the horizontal line passing through the nose keypoint by taking the upper areas as eyes area and the lower area as mouth area. Stimulus videos were reconstructed by overlaying detected body and face segments onto frames and watched to ensure manually that the automatic methods provided a reliable estimation of AOIs. 96.2% of frames across two video episodes (the total number of frames was 61,724) depicted at least one person. Face and face part (eyes and mouth) areas were detected in 94.9% of all frames.

To compute the percentage of total gaze time to an AOI, gaze data were coded into various areas, including faces, eyes, mouth, hands, non-head body, and non-social content, which were defined using the above automated segmentation methods. To perform this coding, first the gaze data were down-sampled to the video frame rate by taking the average of gaze points corresponding to each frame (i.e., there is one average gaze point per frame). Next, the average gaze point was represented as a disk of diameter of 1-degree visual angle. Finally, the gaze was assigned to an AOI based on the maximum area of overlap between a gaze point disk and any of the AOIs considered (see Additional file 1: Fig. S1 E, F).

While computing the percentage of total on-screen gaze time, the number of frames where the gaze fell successfully onto the stimulus presentation monitor was divided by the total number of frames in the video. However, while computing the percentage of total gaze time to a particular AOI, the number of frames where gaze fell onto that area was divided by the total number of frames with successful on-screen gaze. This approach allowed us to control for possible individual differences between participants in their total on-screen gaze time, such that this did not bias the differences in their total gaze time to other AOIs (see “Partial correlation analysis” for additional controls).

### Gaze heatmaps

Temporally binned heatmaps were used to assess the similarity of each participant’s gaze pattern to a reference group (reference group calculations were always leave-one-out, i.e., excluded the comparison participant, if that participant was a member of the respective reference group, to eliminate bias). A gaze heatmap was built for each participant for each time bin by convolving each gaze point in the time bin with a two-dimensional isotropic Gaussian with a standard deviation of 0.5° of visual angle. One degree of visual angle corresponded to 41 square pixels on the screen in our study. A time bin duration of 1 s was used for the results presented here. Other tested time bins (0.5 and 2 s) yielded essentially the same results. A reference gaze heatmap was built for each time bin by applying the same Gaussian convolution on aggregate gaze data from all participants in a group (TD for the TD reference heatmap; ASD for the ASD reference heatmap; see Fig. 1D). The reference gaze heatmaps thus reflected the visual salience of the videos for that participant group (both TD reference and ASD reference heatmaps were highly correlated; see Results). To estimate how an individual’s gaze aligned with a reference visual salience at each time bin, we calculated the correlation between the individual’s gaze heatmap and the reference heatmap for each time bin. To calculate correlation coefficients between heatmaps, each 2-D heatmap was first converted to a single vector of values, and then the Pearson correlation between these two vectors was calculated. One autistic and one control participant exhibiting mean gaze heatmap correlation values exceeding 4 SD from the mean of all other participants were excluded from analyses to produce a more normal distribution for the estimation of effect sizes in group differences (but results remain essentially unchanged with their inclusion).

### Estimation of effect sizes in group differences

Cohen’s *d* was used to provide a standardized measure of the effect size for the difference between the mean values of ASD and TD groups for their gaze time to various features of videos. We preferred Cohen’s *d* to provide a practical estimate for the magnitude of an effect of interest (i.e., difference between groups) rather than just reporting the results of statistical tests that measure the significance of a difference between group means [54, 55]. A bootstrap procedure was used to robustly estimate Cohen’s *d* and its confidence interval. Individual participant’s gaze time percentages or heatmap correlation values were randomly sampled 10,000 times with replacement separately for each group, and Cohen’s *d* between groups was computed at each iteration. The mean Cohen’s *d* for each gaze feature was obtained by averaging the d values across bootstrap iterations. The confidence interval of Cohen’s *d* was taken as 2.5th and 97.5th percentiles of the bootstrap distribution.

To examine the effect of reducing the number of data samples on estimated group differences, we combined this bootstrap resampling of participants with a random sampling of different duration epochs from videos. In this procedure, we first combined gaze data from the two video episodes into a single 43-min dataset. Then (i) an epoch of a specific duration (e.g., 10 min., contiguous) was selected randomly from the combined 43-min data; (ii) 48 autistic and 105 control participants were sampled with replacement (i.e., bootstrap resampling); (iii) Cohen’s *d* was computed for each considered gaze feature (e.g., gaze time to faces) based on gaze data for this selected video epoch and these sampled participants. These steps (i–iii) were repeated 10,000 times to obtain a distribution of Cohen’s *d* for each considered gaze feature. Finally, (iv) mean Cohen’s *d* for each gaze feature was obtained by averaging the d values across 10,000 iterations. The steps (i–iv) were repeated separately for different epoch durations examined (e.g., 10 min, 5 min, 2 min, etc.; see Additional file 1: Table S1).

### Estimation of effect sizes in within-individual reliability

Spearman’s correlation in individuals’ gaze time to various AOIs between two video episodes was used to measure the within-individual reliability in gaze patterns. To obtain a statistically more reliable estimate of the correlation and to examine the effect of data size on the estimated values, we used a random sampling of different duration epochs from videos. In this procedure, we first combined gaze data from the two video episodes into a single 43-min dataset. Then (i) two non-overlapping epochs, each with the same specific duration (e.g., 10 contiguous minutes), were selected randomly from the combined 43-min data; (ii) Spearman’s correlation was computed for each considered gaze feature (e.g., gaze time to faces) based on gaze data from these two epochs. The steps (i–ii) were repeated 10,000 times to obtain a distribution of Spearman’s correlation for each considered gaze feature. Finally, (iii) the mean correlation for each gaze feature was obtained by averaging the correlation values across 10,000 iterations. The steps (i–iii) were repeated separately for different epoch durations examined (see Additional file 1: Table S2).

### Partial correlation analysis

We performed a partial correlation analysis to examine whether the reliability measured in the percentage of gaze time to faces or eyes, or the heatmap correlations with TD reference heatmaps can be explained solely by individual differences between participants in their percentage of on-screen gaze time. This analysis procedure measures the correlation between two variables *x* (i.e., the percentage of gaze time to faces in Episode A) and *y* (i.e., the percentage of gaze time to faces in Episode B) while partialing out the effect of a third variable *z* (i.e., the percentage of on-screen gaze time in Episode A) on *x* and of a fourth variable *w* (i.e., the percentage of on-screen gaze time in Episode B) on *y*. To implement this analysis with the Spearman correlation, first the values of each variable were transformed to their rank scores. Second, to partial out the effect of *z* from *x*, a simple linear regression of *x* on *z* was computed, and the residuals were obtained. The residuals provided the variance in *x* that could not be explained by *z*. Third, the same procedure was applied to obtain the residuals from the regression of *y* on *w*. Finally, the Pearson correlation coefficient (not the Spearman correlation, as the data were already rank-transformed) between the two residuals was computed.

### Gaze fingerprinting

To estimate whether an individual’s patterns of gaze to visual features were reliable and individually distinctive across different epochs of videos, we used a gaze fingerprinting approach [28, 56]. In this approach, each individual’s gaze data within a time bin was represented as an eight-dimensional gaze vector (the percentage of time spent looking at screen, faces, non-social content, non-head body parts, hands, eyes, mouth, and heatmap correlations with TD reference heatmaps). In the identification procedure, given a gaze vector of a participant in a time bin, we calculated the pairwise L2 distances between this gaze vector and all gaze vectors from all participants (including themselves) from another time bin. Participant identity was then predicted based on the minimum pairwise distance. This procedure was repeated for each individual and the successful identifications were counted. Prior to computing distances, each feature channel in the gaze vectors was standardized to zero mean and unit variance across participants within each time bin to control for range and variance differences between features. The statistical significance of identification accuracy was computed by a permutation test with 10,000 iterations. At each iteration of the permutation, identities were shuffled across gaze vectors, and then the fraction of successful identifications was computed. The obtained empirical null distribution of identification accuracy was used to assess the *p* value of the actual measured identification accuracy.

The initial analysis of gaze fingerprinting was performed across the two video episodes. To provide a statistically more reliable estimate of the identification accuracy, we used a random sampling of different duration epochs from videos and a bootstrap resampling of participants. In this procedure, we first combined gaze data from the two video episodes into a single 43-min dataset. Then (i) two non-overlapping epochs, each with the same specific duration (e.g., 10 min., contiguous), were selected randomly from the combined 43-min data; (ii) 48 autistic and 105 control participants were sampled with replacement; (iii) the identification accuracy was computed based on gaze data from these two epochs. The steps (i–iii) were repeated 10,000 times to obtain a distribution of identification accuracy. Finally, (iv) the mean accuracy was obtained by averaging the accuracy values across 10,000 iterations. The steps (i–iv) were also repeated separately for different epoch durations examined (see Additional file 1: Table S6).

### Gaussian mixture model

For an unsupervised partitioning of participants into subgroups, we trained a variational Bayesian Gaussian mixture model [57]. This approach allowed us to learn the number of subgroups (i.e., clusters) from data automatically. To obtain multiple estimates of the number of clusters over different epochs of videos, we first combined gaze data from the two video episodes into a single 43-min dataset. Using a moving window of 10-min length with a step size of 1 min, the gaze data were sampled 33 times. At each epoch, each participant was represented by a four-dimensional vector, where the dimensions are the percentage of total gaze time on-screen, faces, eyes, and average heatmap correlation with TD reference gaze heatmaps as used in previous analyses. These participant-wise four-dimensional vectors were concatenated across participants to obtain a two-dimensional matrix with dimensions (number of participants × 4) for each epoch. A Bayesian Gaussian mixture model was fit to this matrix to reveal underlying clusters based on gaze similarities between participants. The model was initialized to identify five clusters (tests with initialization of 10 or 20 clusters yielded the same results) but returned predominantly two large clusters (each containing more than 30 participants) and three small clusters (each containing one, two, or three participants only). Individuals in these three small clusters were then assigned to one of the large clusters based on the minimum Euclidean distance between an individual’s four-dimensional gaze vector and the mean gaze vector representing the cluster center. To perform this analysis, we used the implementation of variational Bayesian Gaussian mixture models in the Python machine learning library scikit-learn [58].

### Summary of methods

We analyzed eye tracking data from a sample of autistic adults and TD controls matched on age-, sex- and full-scale IQ (Table 1). To best distinguish our three hypotheses, we aimed to assess each participant’s reliability in gaze most precisely. For this reason, we incorporated the following features into our study. First, we selected adult participants with intellectual functioning in the normal range, reducing variation related to general inattention, difficulty following instructions, or difficulty understanding the video stimuli. Second, we used a rich and highly social television sitcom (“The Office”; NBC Universal) as these are the stimuli that we have found successful in probing atypical cognition in our prior neuroimaging work on ASD [43, 59]. Third, we used two different video episodes to test within-individual reliability across stimuli.

## Results

### Visual engagement to social features

We first quantified gaze differences between ASD and TD control groups by calculating the percentage of time spent looking at several predefined visual features in the videos, such as faces, hands, and face parts (see Automatic segmentation of frames to areas of interest in Methods). As expected, compared to the TD group, the ASD group looked less at faces (percentage of face looking time = 77.4% ± 5.8% in Episode A and 70.8% ± 5.6% in Episode B for the ASD group, 80.7% ± 3.8% in Episode A and 73.9% ± 4.2% in Episode B for the TD group, mean ± standard deviation across participants; Fig. 1A and 1B) and less at eyes within faces (percentage of eyes looking time = 52.0% ± 13.0% in Episode A and 47.0% ± 14.2% in Episode B for the ASD group, 60.2% ± 13.3% in Episode A and 54.9% ± 12.3% in Episode B for the TD group; Cohen’s *d* = 0.618 in Episode A and *d* = 0.604 in Episode B for faces, *p* < 0.001, bootstrap test; Fig. 1C), but looked more at other body parts and at non-social content (inanimate objects and/or background).

This AOI-based analysis was complemented by an alternative analysis using gaze heatmaps. The Pearson correlation was used to compare each participant’s gaze heatmap to a TD reference heatmap, both were constructed using gaze data from 1-s epochs of videos (a leave-one-participant-out approach was used to prevent bias for TD individuals, see Gaze heatmaps in Methods). The reference heatmap reflected the visual salience of the videos in respective time bins. We found that the correlation was significantly higher in the TD group than in the ASD group (Pearson’s *r* = 0.61 ± 0.08 in Episode A and 0.59 ± 0.08 in Episode B for the ASD group, 0.67 ± 0.08 in Episode A and 64.8 ± 0.09 in Episode B for the TD group, mean ± standard deviation across participants; Cohen’s *d* = 0.820 in Episode A, *d* = 0.692 in Episode B, *p* < 0.001, bootstrap test; Fig. 1D).

As a complementary analysis, we constructed reference heatmaps from all ASD groups (using a leave-one-out approach for autistic individuals). We found that the reference heatmap based on the ASD group was highly correlated with the reference heatmap based on the TD controls (Pearson’s r between heatmaps = 0.966 ± 0.023 in Episode A; 0.963 ± 0.026 in Episode B, mean ± standard deviation across 1-s time bins within each episode). This result suggests that the shared preferential attention to stimuli between the autistic individuals was highly correlated with that between the TD individuals—even though the prior results showed that they were individually atypical. In addition, the TD group was still more strongly correlated with this new reference heatmap than the ASD group (Pearson’s *r* = 0.60 ± 0.07 in Episode A and 0.58 ± 0.07 in Episode B for the ASD group, 0.66 ± 0.07 in Episode A and 63.4 ± 0.08 in Episode B for the TD group, mean ± standard deviation across participants; Cohen’s *d* = 0.787 in Episode A, *d* = 0.642 in Episode B, *p* < 0.001, bootstrap test; Fig. 1D), corroborating the above result obtained using TD reference heatmaps. The latter result indicates a lack of common gaze pattern among autistic individuals—otherwise the ASD group would have been more strongly correlated with the ASD reference heatmap than the TD group was. These findings thus already provide initial evidence against hypothesis 3 that atypical gaze patterns are homogeneous among autistic individuals (we provide stronger evidence below).

The exact proportion of time spent looking at any feature would be idiosyncratic for any video, depending on exactly what it depicts. We note that overall time spent looking at faces was significantly higher in Episode A than Episode B within each group (Cohen’s *d* = 1.149 in the ASD group and *d* = 1.696 in the TD group, *p* < 0.001, bootstrap test, Fig. 1A). However, the differences between ASD and TD groups in their gaze time to various features of videos remained similar across the two episodes (Fig. 1C). A statistically more robust analysis based on the sampling of different duration epochs from videos and bootstrap resampling of individual participants was used to examine the effect of reducing data size and different epochs on assessed group differences. We found that the significant group differences can be observed with epochs sampled from the video that are as short as 2 min (Additional file 1: Table S1).

### Within-individual reliability

We next examined whether the discriminating differences between the groups (Fig. 1C, D) were driven by noisier (less reliable) gaze patterns in the ASD group (i.e., hypothesis 1). We reasoned that if autistic people have less reliable gaze patterns, then the rank order among autistic individuals in their gaze time to various AOIs (such as faces in the video) as well as rank order of heatmap correlations with TD reference heatmaps should reflect this variability when comparing within-individual data between two sessions (two distinct videos, Episode A and B). An initial test comparing rank-order correlations (Spearman’s *ρ*) between two different video episodes showed that this prediction was incorrect: those participants who look least at specific features (e.g., faces) in one video, also do so reliably in a second video (Fig. 2).

A statistically more robust analysis based on the sampling of 10-min epochs from combined data of two episodes (see Methods) confirmed equivalent and high within-individual reliability in the ASD and TD groups (all correlations significantly different from zero; Spearman’s *ρ* > 0.635, *p* < 0.001, bootstrap test, FDR corrected, Fig. 2C, F, I, and L). Furthermore, the reliability estimates did not differ between the groups (*p* > 0.333 for gaze time to on-screen, faces, eyes, and heatmap correlation; bootstrap test, FDR corrected). In an additional analysis, we examined the reliability values using different duration video epochs in a range of 10 min–0 s (Additional file 1: Table S2). We found that although the estimated reliability values decreased with decreasing epoch duration, as would be expected, there was no significant difference between the values assessed for ASD and TD groups in any of the epoch durations examined (*p* > 0.333, bootstrap test; Additional file 1: Table S2). These results indicate that the gaze patterns of autistic individuals are as reliable as those of TD controls, providing evidence against hypothesis 1.

A potential concern about Fig. 2 is that participants’ on-screen gaze time (panels A-C) could reflect their attention to the task, or the level of visual engagement, and thus large individual differences in this measure might drive high within-individual reliability values estimated for other measures (panels D–L). To examine this issue, we compared the rank-order correlations provided in Fig. 2 with the residual correlations computed after partialing out the effect of on-screen gaze time from gaze time to faces or eyes, or heatmap correlations (Additional file 1: Table S3). This control analysis confirmed that the correlation values shown in Fig. 2 remain essentially unchanged after controlling for individual differences in on-screen gaze time.

Next, we examined whether there is a systematic relation between autism symptom severity and measured gaze patterns in the ASD group. To do this, we calculated Spearman’s rank-order correlations between four gaze features (percentage of on-screen, face- and eye-looking time, and heatmap correlations with TD reference heatmap) and a severity measure (calibrated severity scores, CSS-Overall, which were generated from the Hus and Lord [48] algorithm). We found that there was no significant correlation between any of these gaze features and the examined severity measure (Additional file 1: Table S4).

Finally, to ensure that familiarity with the stimuli did not influence our results, we used questionnaire data that were collected to measure each participant’s prior familiarity with each of the two episodes (ratings in a range from 1 to 9; with 1 meaning “not at all” and 9 meaning “very much”). Splitting participants into those who were familiar (ratings > 5) or unfamiliar (rating < 5) verified that there were no significant differences in any of our reported results between familiar and unfamiliar groups, either when confined to the ASD group or to the TD group (two-tailed, unpaired *t* test, |*t*|< 1.85 and *p* > 0.07; see Additional file 1: Table S5).

### Gaze fingerprinting

To corroborate these findings, we asked whether individuals can be uniquely and reliably distinguished from other participants based on their patterns of gaze alone. For this analysis, we used a multivariate “gaze fingerprinting” procedure [28, 56], which complemented the univariate approach used above in investigating within-individual reliability. We examined the identification of individuals based on the similarity between their gaze patterns across two episodes, quantified using the distribution over eight gaze features: the percentage of time spent looking at the screen, faces, non-social content, non-head body parts, hands, eyes, mouth, and heatmap correlations with TD reference heatmaps. This analysis provided a fingerprinting identification accuracy of 35.4% (17/48) and 29.5% (31/105) for the ASD and TD groups, respectively, both significantly above chance (*p* < 0.001, permutation test; the chance level was 6.2% (3/48) and 2.9% (3/105) from the 95th percentile of an empirical null distribution). A statistically more robust analysis based on the sampling of 10-min epochs from two episodes’ combined data and bootstrap resampling of individual participants (see Methods) provided similar accuracies (34.4% for ASD and 35.6% for TD group; *p* < 0.001, permutation test, FDR corrected). Furthermore, the groups did not differ in identification accuracy (mean[ASD-TD] = 1.2%, *p* = 0.982, bootstrap test).

To test how fingerprinting identification accuracy scales with duration of the video, we gradually reduced the sampling epoch and found that the identification accuracy for each group remained significantly higher than chance for 2-min epochs (20.4%, *p* = 0.013 for ASD and 18.9%, *p* < 0.001 for TD group), but dropped to close to chance level for 1-min epochs (15.5%, *p* = 0.042 for ASD and 14.0%, *p* = 0.029 for TD group, see Additional file 1: Table S6). Next, we examined whether a subset of the eight gaze features might carry sufficient information for fingerprinting as effectively as the full set. We removed gaze time to non-social content, non-head body parts, and hand areas from the feature set because they are highly correlated with gaze time to face areas. We also removed gaze time to mouth areas because of its high correlation with that to eye areas. Gaze fingerprinting analysis based on the four remaining features (percentage of on-screen, face- and eye-looking time, and heatmap correlations) yielded nearly the same identification accuracy as the full set of features (32.6%, *p* < 0.001 for ASD and 35.2%, *p* < 0.001 for TD group for 10-min epochs; 20.6%, *p* = 0.011 for ASD and 21.0%, p < 0.001 for TD group for 2-min epochs). Furthermore, none of these additional analyses revealed any significant difference between the identification accuracy estimated for ASD and TD groups (*p* > 0.896, bootstrap test), consistent with our initial rank-order correlation analyses. Taken together, these findings demonstrate substantial within-individual reliability in gaze that is equivalent in ASD and TD groups, and that is distributed across multiple visual features.

### Within-group heterogeneity

The results shown in Figs. 1 and 2 confirm atypical gaze patterns in autistic individuals and substantial within-individual reliability despite considerable between-individual variability: some individuals always gaze at faces, while some rarely do, yet reliably so. Supporting our hypothesis 2 that atypical gaze patterns are individually reliable but heterogeneous across autistic individuals, this pattern of results provides initial evidence against hypothesis 3 that atypical gaze patterns are individually reliable and also homogeneous among autistic individuals. Furthermore, the fact that autistic individuals are less correlated with the ASD group reference heatmap than individual controls (Fig. 1D) already suggested that the ASD group must be heterogeneous in their gaze patterns. To compare in further detail whether individually reliable gaze patterns are heterogeneous (hypothesis 2) or homogeneous (hypothesis 3) across the ASD group compared to the control group, we performed two complementary analyses.

In the first analysis, we examined the supervised dichotomous classification of ASD versus TD based on their gaze patterns. We reasoned that if atypical gaze patterns are homogeneous across the ASD group, then the classification of individuals as ASD versus TD should be accurate and reliable. We used the Gaussian Naive Bayes algorithm to build a classifier of ASD versus TD groups. To robustly estimate classification accuracy, we sampled a 10-min epoch from the combined data of two episodes, randomly held-out 16 autistic and 35 control individuals (one-third of participants in each group), and trained a classifier on the remaining participants’ data using four gaze features: the percentage of on-screen, face- and eye-looking time, and average heatmap correlations with TD reference heatmaps. As the fingerprinting procedure above indicated, individuals can be reliably identified from their gaze features based on 10-min gaze data using these four gaze features. We then assessed the classification accuracy based on data from held-out participants. This cross-validation (CV) procedure consisting of a random sampling of an epoch and model estimation steps was repeated 10,000 times. We found that the mean classification accuracy was only slightly greater than would be expected by chance (0.618 ± 0.058; mean ± SD across CV iterations; *p* = 0.039, bootstrap test, the chance level was 0.607 from the 95th percentile of an empirical null distribution). To provide insight into the classification accuracy, we examined the frequency of correct classification and misclassification of individual participants across 10,000 CV iterations of the classifier. We found that 95 participants, 15 autistic, were correctly classified more than 75% of the time across CV iterations. However, 32 participants, 22 autistic, were misclassified more than 75% of the iterations (see Fig. 3A). This low classification performance confirms that the ASD group has a heterogeneous gaze pattern, which precludes diagnostically robust classification.

In the second analysis, we examined the unsupervised assignment of individuals into subgroups based on their gaze patterns. We reasoned that if atypical gaze patterns are homogeneous (hypothesis 3) across the ASD group, then discovered subgroups should reliably split autistic individuals from TD controls. We used a Gaussian mixture model to automatically assign participants into subgroups based on the same four gaze features used in the classification analysis. Using a moving window of 10-min epochs across two episodes’ combined data with a step size of 1 min, the number of subgroups was learned from data for 33 separate iterations. This procedure discovered predominantly two subgroups across the iterations (two subgroups 25 times; three subgroups eight times, but with the size of one participant six times, two once, and three once). After combining these small subgroups with the larger two subgroups based on their gaze similarity with the average gaze pattern of larger groups (see Methods), the procedure identified one larger group (Cluster 1; *n* = 103.72 ± 8.71 members, mean ± SD across 33 iterations; 25.48 ± 3.24 autistic) and one smaller group (Cluster 2; *n* = 49.27 ± 8.71 members; 22.52 ± 3.24 autistic). Eighty-eight participants, 20 autistic, were assigned to Cluster 1 for more than 75% of the time across iterations; and 34 participants, 17 autistic, were assigned to Cluster 2 more than 75% of the iterations (Fig. 3B). These numbers suggest that Cluster 1 captured largely TD controls whereas Cluster 2 captured those autistic and TD individuals who deviated from the majority of TD controls. Thus, the clustering result corroborated the prior classification result that the autistic individuals cannot be regarded as a homogeneous group, but rather consisted of at least two separate subgroups, some of them reliably similar to TD controls, and others reliably dissimilar.

Taken together, the classification and clustering results indicate that gaze patterns of autistic people are strongly heterogeneous across individuals and could not be considered as a homogeneous group distinct from TD controls, rejecting hypothesis 3 in favor of hypothesis 2.

## Discussion

Collecting eye tracking data across two 20-min sitcom episodes allowed us to quantify in detail how people look at social and non-social features in videos. There were large individual differences, both in ASD group and in TD controls, in how people looked at the various features in these video stimuli. However, across the two videos (as well as randomly subsampled epochs from them), each individual participant’s gaze pattern was remarkably consistent. Those participants who spent the most time gazing at faces in Episode A of the videos also spent the most time gazing at faces in Episode B, for instance. AOI-based analyses were corroborated by data-driven heatmap analyses. We found that within-individual reliability in how participants looked at video stimuli was remarkably high, even though there were large individual differences across individuals.

Given that both autistic and control participants were individually reliable in their gaze patterns to videos, we next asked whether this reliability could be used to identify individuals across different epochs of videos at a level that is better than would be expected by chance. Using a fingerprinting analysis, which is sensitive to both within-individual reliability and between-individual distinctiveness, we indeed found this to be the case. These findings thus eliminate our hypothesis 1 that gaze patterns of autistic individuals are less reliable or noisier than those of controls, but still leave open hypothesis 2 that atypical gaze patterns are individually reliable but heterogeneous across ASD participants or hypothesis 3 that atypical gaze patterns are individually reliable and also homogeneous among autistic people. The large between-individual differences seen in both participant groups already suggest that hypothesis 3 is unlikely to be the case. However, we tested this further with classification methods.

We next used both supervised and unsupervised classification methods to ask whether the participant group (ASD or TD) could be reliably identified from an individual’s gaze pattern. We found this not to be the case: both classification approaches had poor accuracy and resulted in many misclassifications. There was also initial evidence suggesting the possible presence of further subtypes of participants as defined by their gaze patterns, a suggestion that will require larger sample sizes to properly assess (see Limitations). Taken together, the findings provide strong support for our hypothesis 2: gaze to naturalistic videos is heterogeneous across autistic participants (large between-individual variability), and this is as reliable from one video to the next as in controls (small within-individual variability).

Are autistic individuals all distinct in their own way, or is there evidence suggesting subgroups? The answer may be: both. Our clustering analysis points to at least two possible subgroups of ASD in the present data: those with gaze similar to TD controls, and a subgroup with heterogeneous but individually reliable atypical gaze. This pattern is reminiscent of neuroimaging results we [43] and others [45] have documented previously, and leaves open the discovery of homogeneous ASD subtypes if larger sample sizes can be analyzed in future studies. The systematic differences in how autistic participants look at visual stimuli compared to TD controls would be expected to translate to differences also in evoked BOLD-fMRI activations [24] and may be an endophenotype reflecting substantial genetic effects [27, 28]. While genetics, gaze, and neuroimaging data will all need to be put together for a comprehensive mechanistic explanation of ASD, eye tracking will continue to have strong practical advantages, given current technological developments affording collection at home from laptop-based [60] or smartphone-based cameras [61]. Such in-home collection could be used in order to achieve the larger sample sizes required to further test both for long-term longitudinal stability within individuals, as well as to explore possible subtypes among them.

The practical advantage of eye tracking data is further borne out by our finding that surprisingly short epochs of the video (2 min) and re-testing after a relatively short time (an hour or so from Episode A to Episode B) were sufficient to produce the patterns we report. The robustness of our findings to these relatively short time windows also suggests that long-timescale semantic features of the videos (the overall arc of the story, for instance) were not features driving the results. With respect to specific features, we found a rather distributed effect over heatmaps and facial features, with no single feature driving the effect. However, it is worth noting that our features were correlated with various degrees and that the videos we used always had social features (people) present. Future studies with more specifically designed visual features could help to further constrain those features that are the most informative and could in principle be used to derive video stimuli with even higher within-individual reliability and between-individual differences. Possibly such stimuli could also improve the classification analyses in distinguishing autistic individuals from controls.

While posing some challenges of their own, naturalistic stimuli such as movies have rapidly become the stimulus of choice for many studies in social neuroscience [62] and for good reason: compared to the much more impoverished and artificial stimuli typically employed in earlier studies (such as cropped photographs of grayscale faces), movies have the benefit of high participant engagement, and reducing confounds to data quality including sleepiness and motion artifacts [63]. In brain imaging studies, they have also been used to advantage in studies of autism, leveraging one broad data-driven class of analyses: inter-subject brain correlations that demonstrate the power of this stimulus to drive shared brain processing in relation to which individual differences can be sensitively detected [43, 45, 64–67]. Ever since early studies that found atypical eye tracking to movies in autistic people [29], a number of studies have found this type of stimulus to be a particularly efficient and sensitive tool. Moreover, there are now highly annotated shared video stimuli available that have been used in several brain imaging and eye tracking studies (e.g., StudyForrest [68]). Eye movements to engaging stimuli are also easily obtainable across the lifespan, from infancy through adulthood, and are ideally suited to inform basic mechanisms of dysfunction in autism while also identifying quantitatively defined subtypes and markers.

## Limitations

There are three main limitations to our study. First, our results are based on a relatively small sample that is not representative of ASD in general. While the broad conclusions about temporal stability of gaze patterns in ASD appear robust across our sample, the power to leverage this individual-level stability to better understand between-individual variability, let alone to discover possible ASD subtypes, is clearly limited. However, this limitation could be easily addressed in future studies, since eye tracking is in principle possible over the Internet (e.g., WebGazer [69]) and even through mobile phones [61], enabling much larger sample sizes. Furthermore, our sample consisted of adults with IQ in the normal range, and it remains unclear if our findings would generalize to children and individuals with lower IQ. Relatedly, we include common comorbidity, such as mild–moderate depression, anxiety, and ADHD in our sample, leaving open questions about the extent to which these may have contributed to our findings.

A second methodological limitation concerns not the number of participants, but the number of distinct blocks of temporal samples. We collected only two, both close in time and for similar (but distinct) video episodes. It is important to note that the precision of our within-individual participant data is of course limited by measurement error. However, it could also be limited by relevant state-dependent changes within an individual. It would be important to collect longer-term longitudinal data, and data across a larger diversity of stimuli to better address this issue. A particularly valuable direction would be to collect longitudinal eye tracking data that incorporate dense state-dependent measures, such as sleepiness and mood across data collection sessions. Such a much more comprehensive inventory could give us a window into how variable visual attention is across weeks or even years, across development and the lifespan in general, and in relation to changes in emotion and other states that may be associated with autism symptom severity. It could also be used to assess the efficacy of interventions, and even provide some insight into what processes the intervention is changing (e.g., gaze to faces but not to other features might change throughout training). These future directions could also be implemented through Internet-based data collection as we mentioned above. The fact that individual samples could be as short as 2-min, given our results, makes this potentially feasible even in individuals with more severe autism, or in infants.

A final limitation concerns the interpretation of our findings. We have interpreted them as evidence for the hypothesis that autistic participants show good test–retest reliability—low variability—within an individual. The reliability we found in autistic participants was comparable to what we found in control participants. However, we only measured eye movements. It remains possible that other dependent measures—performance tasks or questionnaires based on interpreting the videos, or neuroimaging—would have revealed higher variability in ASD. One version of this limitation is the possibility that perhaps autistic people might have atypically variable cognition over time (i.e., unreliable cognition) but that they are able to compensate for this, at least in our sample of individuals with IQ in the normal range, to produce relatively stable gaze patterns. What makes this possibility less likely is the fact that eye movements are not under strong volitional control, and that the stimuli we used are highly engaging. A top-down compensatory mechanism thus seems implausible.

## Conclusions

We asked whether autistic people show variability in their gaze patterns at the within-individual level (individual reliability) and/or the between-individual level. Using eye tracking across two videos, and analyzing data across two performance sites, we quantified within-individual and between-individual variability in gaze patterns in videos. Taken together, the classification and clustering results indicate that gaze patterns of autistic people are strongly heterogeneous across individuals and could not be considered as a homogeneous group distinct from TD controls, rejecting hypothesis 3 that atypical gaze patterns are homogeneous among autistic individuals in favor of hypothesis 2 that the gaze patterns are heterogeneous across autistic individuals. The strong cross-video correlations in gaze patterns, together with the fingerprinting analysis, demonstrate high individual reliability, rejecting hypothesis 1 that gaze patterns of autistic individuals are less reliable or noisier than those of controls, and instead also supporting hypothesis 2: atypical gaze patterns are heterogeneous between autistic individuals but reliable within an individual.

## Supplementary Information

**Additional file1**. **Figure S1:** Automatic segmentation of frames to areas of interest (AOIs). **A** Because of the copyright restrictions of the sitcom “The Office”, the visualization is shown by using a sample royalty free image (by Alena Darmel from Pexels.com). **B** Automatic segmentation of video frames to detect regions depicting human body parts, including head (yellow), hands (pink), and other body parts (blue). Remaining non-shaded areas (i.e., black areas in panel D) were taken as non-social context. **C** Automatic segmentation of frames to detect regions depicting human faces and estimation of five facial keypoints, including two eyes, nose, and two sides of the mouth. These keypoints were used to define eyes (orange) and mouth (turquoise) areas within each frame. D Segmentation results from panels B and C are combined. **E** A sample gaze point is shown as a disk of diameter of 1-degree visual angle. **F** The gaze point was combined with AOIs to determine the gaze was onto which AOI. **Table S1**: Effect size of the differences (quantified with Cohen’s *d*) between groups in their percentage of total gaze time on-screen, faces, eyes, and in their average heatmap correlation with TD reference gaze heatmaps. Cohen’s *d* between the groups (TD-ASD) were computed within randomly sampled epochs of videos (duration given in rows, see Methods) for 10,000 iterations and then averaged across the iterations. Values in parentheses show the statistical significance of effect size (bootstrap test, FDR corrected for multiple comparisons within each epoch duration). This table complements Fig. 1C, D using a sampling procedure that examines the effect of reducing data size on estimated group differences. Asterisk denotes *p* < 0.001. **Table S2**: Spearman’s correlation among individuals within a group in their gaze time to various AOIs and in their average gaze heatmap correlation with TD reference heatmaps. Correlation values were computed between two randomly sampled epochs of videos (duration given in columns) for 10,000 sampling iterations and then averaged across the iterations. Values in parentheses show the statistical significance of the correlation (bootstrap test, FDR corrected for multiple comparisons within each epoch duration). This table complements analyses provided in Fig. 2C, F, I, L for different duration sampling epochs. Asterisk denotes *p* < 0.001. **Table S3**: In the first row (“ASD - Gaze to faces”), Corr(XEpA, XEpB) reports Spearman’s correlation (and its statistical significance, asterisk denoting *p* < 0.001) between data from two separate videos (Episode A and B) for the percentage of gaze time to faces for the ASD group (as shown in Fig. 2A). Corr(XEpA, On-ScreenEpA) reports the correlation between on-screen and face gaze times in Episode A for the group. ParCorr(XEpA, XEpB) reports the residual (partial) correlation between gaze times to faces in Episode A and B after partialing out the effect of on-screen gaze time from gaze time to faces separately in each episode. Other rows in the table repeat this analysis for other gaze features and TD group. **Table S4**: Spearman’s correlation (and its statistical significance, uncorrected) between four gaze features (percentage of on-screen, face- and eye-looking time, and heatmap correlations with TD reference heatmaps) and an autism severity measure (calibrated severity scores, CSS-Overall, which were generated from the Hus and Lord algorithm; see main text) in Episode A (EpA) and Episode B (EpB). **Table S5**: Effect of familiarity with an episode on gaze features. A 9-point questionnaire (in a range from 1 to 9) about prior familiarity with each episode was used to split participants into those who were familiar (ratings > 5) or unfamiliar (ratings < 5) with an episode. The calculated t-statistic (and its p value) for the means of familiar and unfamiliar participants in the ASD group, in the TD group, and across all participants independent of ASD diagnosis for their four gaze features (percentage of on-screen, face- and eye-looking time, or average heatmap correlations with TD reference heatmaps). T tests were two-tailed and unpaired, assuming equal variance. For Episode A (EpA), 17 (24) autistic individuals and 50 (47) TD controls were familiar (unfamiliar) with the episode. For Episode B (EpB), 13 (32) autistic individuals and 39 (52) TD controls were familiar (unfamiliar) with the episode. **Table S6**: The change in fingerprinting identification accuracy as a function of sampling epoch duration. The fingerprinting analysis was performed either using eight gaze features (the percentage of time spent looking at screen, faces, non-social content, non-head body parts, hands, eyes, mouth, and heatmap correlations with TD reference heatmaps) or four features (percentage of on-screen, face- and eye-looking time, heatmap correlations). Accuracy values were computed using two randomly sampled epochs of videos (duration given in columns) for 10,000 times and then averaged across the iterations. Values in parentheses show the statistical significance of accuracy (bootstrap test, FDR corrected for multiple comparisons within each epoch duration). Asterisk denotes *p* < 0.001.

## Data Availability

All data and code can be publicly accessed at https://github.com/adolphslab/adolphslab_eyetracking.

## Abbreviations

ASD: Autism spectrum disorder participant group
TD: Typically developing participant group
ADOS: Autism Diagnostic Observation Schedule
AOI: Area of interest
IU: Indiana University
SD: Standard deviation
CI: Confidence interval
CV: Cross-validation
