# Supplementary material for: Atypical gaze patterns in autistic adults are heterogeneous across but reliable within individuals
Source: Mol Autism. 2022 Sep 24;13:39. doi: 10.1186/s13229-022-00517-2 (PMC9508778; doi:10.1186/s13229-022-00517-2)
Supplement: Supplementary file 1 — Additional file1. Figure S1: Automatic segmentation of frames to areas of interest (AOIs). A Because of the copyright restrictions of the sitcom “The Office”, the visualization is shown by using a sample royalty free image (by Alena Darmel from Pexels.com). B Automatic segmentation of video frames to detect regions depicting human body parts, including head (yellow), hands (pink), and other body parts (blue). Remaining non-shaded areas (i.e., black areas in panel D) were taken as non-social context. C Automatic segmentation of frames to detect regions depicting human faces and estimation of five facial keypoints, including two eyes, nose, and two sides of the mouth. These keypoints were used to define eyes (orange) and mouth (turquoise) areas within each frame. D Segmentation results from panels B and C are combined. E A sample gaze point is shown as a disk of diameter of 1-degree visual angle. F The gaze point was combined with AOIs to determine the gaze was onto which AOI. Table S1: Effect size of the differences (quantified with Cohen’s d) between groups in their percentage of total gaze time on-screen, faces, eyes, and in their average heatmap correlation with TD reference gaze heatmaps. Cohen’s d between the groups (TD-ASD) were computed within randomly sampled epochs of videos (duration given in rows, see Methods) for 10,000 iterations and then averaged across the iterations. Values in parentheses show the statistical significance of effect size (bootstrap test, FDR corrected for multiple comparisons within each epoch duration). This table complements Fig. 1C, D using a sampling procedure that examines the effect of reducing data size on estimated group differences. Asterisk denotes p < 0.001. Table S2: Spearman’s correlation among individuals within a group in their gaze time to various AOIs and in their average gaze heatmap correlation with TD reference heatmaps. Correlation values were computed between two randomly sampled epochs of videos (duration given i [file 13229_2022_517_MOESM1_ESM.docx]

**Supplementary Information**


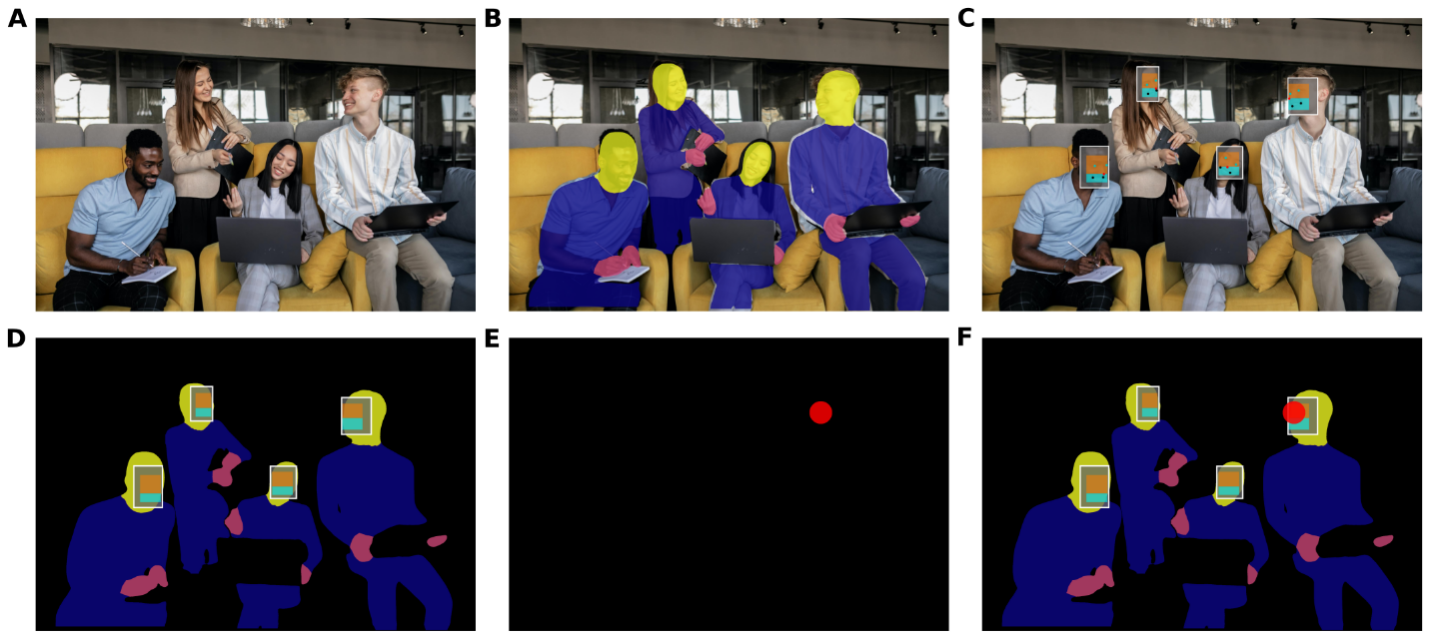


Fig. S1. Automatic segmentation of frames to areas of interest (AOIs). **A** Because of the copyright restrictions of the sitcom “The Office”, the visualization is shown by using a sample royalty free image (by Alena Darmel from Pexels.com). **B** Automatic segmentation of video frames to detect regions depicting human body parts, including head (yellow), hands (pink), and other body parts (blue). Remaining non-shaded areas (i.e., black areas in panel D) were taken as non-social context. **C** Automatic segmentation of frames to detect regions depicting human faces and estimation of five facial keypoints, including two eyes, nose, and two sides of the mouth. These keypoints were used to define eyes (orange) and mouth (turquoise) areas within each frame. **D** Segmentation results from panels B and C are combined. **E** A sample gaze point is shown as a disk of diameter of 1-degree visual angle. **F** The gaze point was combined with AOIs to determine the gaze was onto which AOI.

|  | Gaze feature | | | |
| --- | --- | --- | --- | --- |
| Epoch duration | On-screen | Faces | Eyes | Heatmap corr. |
| 10 mins | 0.493 (0.010) | 0.678 (0.001) | 0.609 (0.001) | 0.747 (*) |
| 5 mins | 0.483 (0.018) | 0.641 (0.004) | 0.590 (0.004) | 0.708 (*) |
| 2 mins | 0.444 (0.045) | 0.571 (0.013) | 0.568 (0.011) | 0.671 (0.006) |
| 1 min | 0.409 (0.073) | 0.495 (0.042) | 0.539 (0.018) | 0.627 (0.016) |
| 30 secs | 0.367 (0.128) | 0.430 (0.126) | 0.503 (0.057) | 0.574 (0.057) |
| 20 secs | 0.347 (0.156) | 0.396 (0.156) | 0.481 (0.084) | 0.545 (0.084) |
| 10 secs | 0.309 (0.228) | 0.336 (0.228) | 0.438 (0.142) | 0.487 (0.142) |

Table S1. Effect size of the differences (quantified with Cohen’s d) between groups in their percentage of total gaze time on-screen, faces, eyes, and in their average heatmap correlation with TD-reference gaze heatmaps. Cohen’s d between the groups (TD-ASD) were computed within randomly sampled epochs of videos (duration given in rows, see Methods) for 10,000 iterations and then averaged across the iterations. Values in parentheses show the statistical significance of effect size (bootstrap test, FDR corrected for multiple comparisons within each epoch duration). This table complements Figs. 1C and 1D using a sampling procedure that examines the effect of reducing data size on estimated group differences. Asterisk denotes *p* < 0.001.

|  |  | Sampling epoch duration | | | | | | |
| --- | --- | --- | --- | --- | --- | --- | --- | --- |
| Gaze feature | Group | 10 mins | 5 mins | 2 mins | 1 min | 30 secs | 20 secs | 10 secs |
| On-screen | ASD | 0.635 (*) | 0.619 (0.002) | 0.553 (0.012) | 0.499 (0.034) | 0.442 (0.096) | 0.408 (0.108) | 0.352 (0.274) |
|  | TD | 0.789 (*) | 0.765 (*) | 0.694 (*) | 0.627 (0.002) | 0.559 (0.018) | 0.518 (0.040) | 0.457 (0.110) |
|  | TD-ASD | 0.154 (0.333) | 0.146 (0.418) | 0.141 (0.605) | 0.129 (0.707) | 0.117 (0.784) | 0.110 (0.843) | 0.105 (0.910) |
| Faces | ASD | 0.685 (*) | 0.642 (*) | 0.529 (0.010) | 0.425 (0.060) | 0.315 (0.192) | 0.261 (0.314) | 0.181 (0.579) |
|  | TD | 0.775 (*) | 0.691 (*) | 0.543 (0.002) | 0.414 (0.036) | 0.300 (0.184) | 0.247 (0.314) | 0.163 (0.579) |
|  | TD-ASD | 0.090 (0.485) | 0.049 (0.780) | 0.014 (0.964) | -0.011 (0.944) | -0.015 (0.940) | -0.014 (0.944) | -0.019 (0.948) |
| Eyes | ASD | 0.784 (*) | 0.738 (*) | 0.670 (0.002) | 0.591 (0.014) | 0.500 (0.094) | 0.450 (0.108) | 0.365 (0.274) |
|  | TD | 0.864 (*) | 0.825 (*) | 0.747 (*) | 0.666 (0.002) | 0.565 (0.018) | 0.499 (0.040) | 0.400 (0.271) |
|  | TD-ASD | 0.080 (0.485) | 0.087 (0.523) | 0.077 (0.678) | 0.075 (0.761) | 0.064 (0.843) | 0.049 (0.910) | 0.035 (0.948) |
| Heatmap corr. | ASD | 0.672 (*) | 0.600 (0.002) | 0.524 (0.016) | 0.451 (0.059) | 0.371 (0.182) | 0.330 (0.259) | 0.256 (0.490) |
|  | TD | 0.759 (*) | 0.723 (*) | 0.649 (*) | 0.568 (0.006) | 0.476 (0.031) | 0.422 (0.069) | 0.331 (0.274) |
|  | TD-ASD | 0.088 (0.485) | 0.122 (0.518) | 0.125 (0.605) | 0.117 (0.707) | 0.105 (0.784) | 0.092 (0.843) | 0.075 (0.924) |

Table S2. Spearman’s correlation among individuals within a group in their gaze time to various AOIs and in their average gaze heatmap correlation with TD-reference heatmaps. Correlation values were computed between two randomly sampled epochs of videos (duration given in columns) for 10,000 sampling iterations and then averaged across the iterations. Values in parentheses show the statistical significance of the correlation (bootstrap test, FDR corrected for multiple comparisons within each epoch duration). This table complements analyses provided in Figs. 2C, 2F, 2I, and 2L for different duration sampling epochs. Asterisk denotes *p* < 0.001.

| Gaze feature (X) | Corr(X_EpA_, X_EpB_) | Corr(X_EpA_,  On-Screen_EpA_) | Corr(X_EpB_,  On-Screen_EpB_) | ParCorr(X_EpA_, X_EpB_) |
| --- | --- | --- | --- | --- |
| ASD - Gaze to faces | 0.733 (*) | 0.082 (0.580) | 0.379 (0.008) | 0.747 (*) |
| ASD - Gaze to eyes | 0.854 (*) | 0.178 (0.226) | 0.236 (0.107) | 0.851 (*) |
| ASD - Heatmap corr. | 0.764 (*) | 0.320 (0.026) | 0.460 (0.001) | 0.737 (*) |
| TD - Gaze to faces | 0.826 (*) | 0.378 (*) | 0.371 (*) | 0.798 (*) |
| TD - Gaze to eyes | 0.880 (*) | 0.151 (0.123) | 0.239 (0.014) | 0.885 (*) |
| TD - Heatmap corr. | 0.802 (*) | 0.144 (0.142) | 0.278 (0.004) | 0.796 (*) |

Table S3. In the first row (“ASD - Gaze to faces”), Corr(X_EpA_, X_EpB_) reports Spearman’s correlation (and its statistical significance, asterisk denoting *p* < 0.001) between data from two separate videos (Episode A and B) for the percentage of gaze time to faces for the ASD group (as shown in Fig. 2A). Corr(X_EpA_, On-Screen_EpA_) reports the correlation between on-screen and face gaze times in Episode A for the group. ParCorr(X_EpA_, X_EpB_) reports the residual (partial) correlation between gaze times to faces in Episode A and B after partialing out the effect of on-screen gaze time from gaze time to faces separately in each episode. Other rows in the table repeat this analysis for other gaze features and TD group.

|  | Gaze feature | | | | | | | |
| --- | --- | --- | --- | --- | --- | --- | --- | --- |
| Score | On-screen EpA | On-screen EpB | Faces EpA | Faces EpB | Eyes EpA | Eyes EpB | Heatmap corr. EpA | Heatmap corr. EpB |
| CSS  Overall | 0.028 (0.851) | -0.091 (0.538) | -0.134 (0.365) | -0.115 (0.437) | 0.053 (0.722) | -0.001 (0.997) | -0.044 (0.769) | -0.039 (0.791) |

Table S4. Spearman’s correlation (and its statistical significance, uncorrected) between four gaze features (percentage of on-screen, face- and eye-looking time, and heatmap correlations with TD-reference heatmaps) and an autism severity measure (calibrated severity scores, CSS-Overall, which were generated from the Hus and Lord algorithm; see main text) in Episode A (EpA) and Episode B (EpB).

|  | Gaze feature | | | | | | | |
| --- | --- | --- | --- | --- | --- | --- | --- | --- |
| Group | Onscreen EpA | Onscreen EpB | Face EpA | Face EpB | Eyes EpA | Eyes EpB | Heatmap corr. EpA | Heatmap corr. EpB |
| ASD  (familiar -  unfamiliar) | 0.025 (0.980) | 0.041 (0.968) | 0.174 (0.863) | -1.672 (0.102) | -0.039 (0.969) | -0.792 (0.433) | -0.240 (0.811) | -0.448 (0.656) |
| TD  (familiar -  unfamiliar) | -0.055 (0.956) | -0.617 (0.539) | 0.346 (0.730) | -1.846 (0.068) | 1.186 (0.238) | 0.029 (0.977) | 0.880 (0.381) | -0.016 (0.987) |
| All  (familiar -  unfamiliar) | 0.187 (0.852) | 0.084 (0.933) | 0.645 (0.520) | -1.761 (0.080) | 1.217 (0.226) | 0.055 (0.957) | 0.955 (0.341) | 0.290 (0.772) |

Table S5. Effect of familiarity with an episode on gaze features. A 9-point questionnaire (in a range from 1 to 9) about prior familiarity with each episode was used to split participants into those who were familiar (ratings > 5) or unfamiliar (ratings < 5) with an episode. The calculated t-statistic (and its p-value) for the means of familiar and unfamiliar participants in the ASD group, in the TD group, and across all participants independent of ASD diagnosis for their four gaze features (percentage of on-screen, face- and eye-looking time, or average heatmap correlations with TD-reference heatmaps). T-tests were two-tailed and unpaired, assuming equal variance. For Episode A (EpA), 17 (24) autistic individuals and 50 (47) TD controls were familiar (unfamiliar) with the episode. For Episode B (EpB), 13 (32) autistic individuals and 39 (52) TD controls were familiar (unfamiliar) with the episode.

|  | 8 gaze features | | | 4 gaze features | | |
| --- | --- | --- | --- | --- | --- | --- |
| Epoch duration | ASD | TD | Difference (TD-ASD) | ASD | TD | Difference (TD-ASD) |
| 10 mins | 34.4% (*) | 35.6% (*) | 1.2% (0.982) | 32.6% (*) | 35.2% (*) | 2.6% (0.896) |
| 5 mins | 28.2% (*) | 27.9% (*) | -0.3% (0.938) | 27.1% (*) | 29.7% (*) | 2.6% (0.902) |
| 2 mins | 20.4% (0.013) | 18.9% (*) | -1.5% (0.983) | 20.6% (0.011) | 21.0% (*) | 0.4% (0.972) |
| 1 min | 15.5% (0.042) | 14.0% (0.029) | -1.5% (0.984) | 15.8% (0.04) | 15.7% (0.011) | -0.2% (0.923) |
| 30 secs | 11.8% (0.139) | 10.6% (0.085) | -1.3% (0.987) | 12.1% (0.152) | 11.6% (0.086) | -0.5% (0.93) |

Table S6. The change in fingerprinting identification accuracy as a function of sampling epoch duration. The fingerprinting analysis was performed either using eight gaze features (the percentage of time spent looking at screen, faces, non-social content, non-head body parts, hands, eyes, mouth, and heatmap correlations with TD-reference heatmaps) or four features (percentage of on-screen, face- and eye-looking time, heatmap correlations). Accuracy values were computed using two randomly sampled epochs of videos (duration given in columns) for 10,000 times and then averaged across the iterations. Values in parentheses show the statistical significance of accuracy (bootstrap test, FDR corrected for multiple comparisons within each epoch duration). Asterisk denotes *p* < 0.001.
